# Malnutrition Is Associated With Worse Outcomes of Inpatient Endoscopic Retrograde Cholangiopancreatography

**DOI:** 10.7759/cureus.26253

**Published:** 2022-06-23

**Authors:** Daniel S Rim, Alexander J Kaye, Weizheng Wang

**Affiliations:** 1 Internal Medicine, Rutgers New Jersey Medical School, Newark, USA; 2 Gastroenterology and Hepatology, Rutgers New Jersey Medical School, Newark, USA

**Keywords:** nutrition status, mortality, outcome analysis, s:malnutrition, endoscopic retrograde cholangiopancreatography (ercp)

## Abstract

Objectives

Endoscopic retrograde cholangiopancreatography (ERCP) is frequently used to manage pancreaticobiliary disorders in an inpatient setting. Malnutrition is prevalent among hospitalized patients, and it is generally associated with poor clinical outcomes. However, there is a lack of studies on how malnutrition affects the outcomes of inpatient ERCP. Thus, we investigated the outcomes of inpatient ERCP among patients with malnutrition.

Methods

Adult patients who underwent ERCP from the 2014 National Inpatient Sample database were selected to conduct retrospective analysis. Patient demographics and outcomes of ERCP were compared between the groups with and without malnutrition. The outcomes of interest were inpatient mortality, length of stay, total hospital charge, and ERCP complications, including pancreatitis, cholecystitis, cholangitis, sepsis, hemorrhage, and intestinal perforation.

Results

Patients with malnutrition had longer length of stay (15.5 days vs. 6.7 days, p < 0.05) and higher total hospital charge ($149,699 vs. $71,723, p < 0.05). Malnutrition was an independent risk factor for inpatient mortality (adjusted odds ratio (aOR) 2.54, 95% confidence interval (CI): 1.70-3.82, p < 0.05), sepsis (aOR 2.20, 95% CI: 1.82-2.65, p < 0.05), hemorrhage (aOR 1.64, 95% CI: 1.05-2.56, p < 0.05), and intestinal perforation (aOR 4.29, 95% Cl:1.61-11.46, p < 0.05).

Conclusions

Our study indicates that patients with malnutrition are more likely to have worse outcomes, such as increased inpatient mortality, sepsis, hemorrhage, and intestinal perforation. Understanding the nutrition status of patients undergoing ERCP can be a useful approach for risk stratification and determining if closer surveillance of the complications is warranted.

## Introduction

Endoscopic retrograde cholangiopancreatography (ERCP) is a widely used procedure for the management of pancreaticobiliary disorders such as choledocholithiasis, acute cholangitis, and acute biliary pancreatitis [1]. Studies estimate that approximately 350,000-500,000 ERCPs are performed every year, and it is performed in about 0.38-0.45% of the total number of discharges from 2007 to 2016 [1,2]. The number of ERCPs performed has been increasing annually [3]. Despite its important role, ERCP has been linked to complications in about 7% of cases [4]. Pancreatitis, infections, hemorrhage, and perforations were more commonly seen, and mortality was present in 0.33% of the cases [4].

Although malnutrition may carry different definitions, it can be defined as a state in which inadequate nutrition intake or uptake causes decreased body mass and impaired physical and mental function [5]. Malnutrition is commonly found among hospitalized patients and may be present in up to 50% of the patients [6]. Malnutrition is generally known to be associated with poor clinical outcomes [7-10]. Previous studies have shown that malnutrition is associated with prolonged length of stay, increased hospitalization cost, and higher mortality [9,10]. In addition, it is associated with adverse effects on the immune system, wound healing, and muscle wasting [8].

Due to the high prevalence of malnutrition in hospitalized patients and widespread use of ERCP, patients who undergo ERCP may have malnutrition, which may complicate their hospital courses, given that malnutrition is linked to worse outcomes in general. However, there is a lack of studies that explicitly address how malnutrition affects the inpatient outcomes of ERCP. Thus, this study aims to assess the outcomes of inpatient ERCP among patients with malnutrition.

This article was previously presented as a meeting abstract at the American College of Gastroenterology (ACG) 2021 Annual Scientific Meeting in October 2021.

## Materials and methods

**Figure 1:**
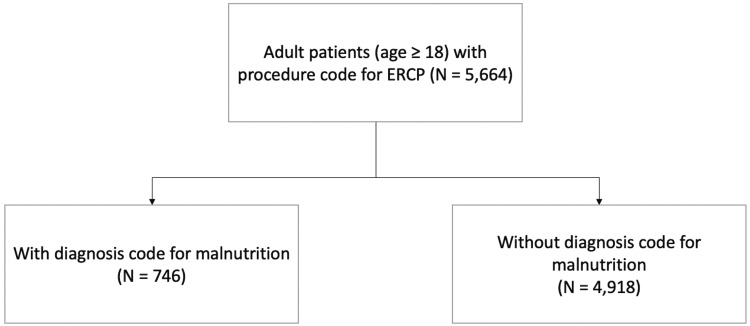
Diagram of study design ERCP, endoscopic retrograde cholangiopancreatography

This is a retrospective cohort study investigating adult patients (aged 18 and above) who underwent ERCP in an inpatient setting. Data from the National Inpatient Sample (NIS), 2014, Healthcare Cost and Utilization Project (HCUP), Agency for Healthcare Research and Quality, which is known as the largest all-payer inpatient database in the United States, were used [11]. The study was conducted at Rutgers New Jersey Medical School in Newark, New Jersey, United States. The International Classification of Diseases-Ninth Edition Revision, Clinical Modification (ICD-9 CM) codes were used to identify diagnoses in this database. Patients were then divided into two groups: those with and without malnutrition (Figure 1). ICD-9 CM codes for various degrees of malnutrition, protein-calorie malnutrition, failure to thrive, and cachexia were used to identify those with malnutrition in this study. Patient demographics and characteristics, including age, sex, race, and the Charlson Comorbidity Index, were compared between the groups. The Charlson Comorbidity Index is a tool frequently used for comorbidity adjustment for confounding [12,13]. The clinical outcomes of ERCP, including inpatient mortality, length of stay, total hospital charge, pancreatitis, cholecystitis, cholangitis, sepsis, hemorrhage, and intestinal perforation, were compared between the groups.

IBM SPSS Statistics for Windows, Version 28.0 (Released 2021; IBM Corp, Armonk, New York, United States) was used for all statistical analyses. Chi-squared tests and independent t-tests were performed to compare proportions and means, respectively. Statistical analyses in this study were two-tailed, and a p-value less than 0.05 was considered statistically significant. To determine if malnutrition is an independent predictor of the clinical outcomes adjusting for age, sex, race, and the Charlson Comorbidity Index, multivariate logistic regression analysis was used.

## Results

Among the 5,664 patients who underwent ERCP, 746 patients had a history of malnutrition. Table 1 demonstrates patient demographics, characteristics, length of stay, total hospital charge, inpatient mortality, and the Charlson Comorbidity Index. Those with malnutrition were older (65.1 years old vs. 59.9 years old, p < 0.05), more likely to be male (51.6% vs. 43.9%, p < 0.05), more likely to be White (66.8% vs. 65.9%, p < 0.05), had longer length of stay (15.5 days vs. 6.7 days, p < 0.05), had higher total hospital charge ($149,699 vs. $71,723, p < 0.05), higher inpatient mortality (5.4% vs. 1.7%, p < 0.05), and higher Charlson Comorbidity Index (4.6 vs. 3.3, p < 0.05).

**Table 1:** Demographics, characteristics, length of stay, total hospital charge, inpatient mortality, and the Charlson Comorbidity Index among patients who underwent ERCP with or without malnutrition *Exact numbers are not included in the table due to small sample sizes.

Variable	With malnutrition	Without malnutrition	p-value
N = 5,664	N = 746	N = 4,918	
Patient age, mean (SD)	65.1 (16.2)	59.9 (18.1)	< 0.05
Sex, N (%)			< 0.05
Female	361 (48.4%)	2760 (56.1%)	
Male	385 (51.6%)	2156 (43.9%)	
Race, N (%)			< 0.05
White	474 (66.8%)	3101 (65.9%)	
Black	107 (15.1%)	508 (10.8%)	
Hispanic	68 (9.6%)	687 (14.6%)	
Asian or Pacific Islander	36 (5.1%)	185 (3.9%)	
Native American	*	31 (0.7%)	
Other	*	191 (4.1%)	
Length of stay, in days (SD)	15.5 (19.8)	6.7 (7.3)	< 0.05
Total hospital charge, in $ (SD)	149,699 (193,542)	71,723 (85,054)	< 0.05
Inpatient mortality	40 (5.4%)	82 (1.7%)	< 0.05
Charlson Comorbidity Index (SD)	4.6 (2.8)	3.3 (2.7)	< 0.05

Table 2 shows clinical outcomes of ERCP in patients with and without a history of malnutrition. Patients with malnutrition had a higher prevalence of cholangitis (27.5% vs. 21.2%, p <0.05), sepsis (31.8% vs. 14.6%, p < 0.05), and hemorrhage (3.8% vs. 2.1%, p <0.05). There were no statistically significant differences in the prevalence of pancreatitis or cholecystitis. Data analysis of intestinal perforation was limited due to the small sample size in the group with malnutrition.

**Table 2:** Clinical outcomes of ERCP with and without a history of malnutrition *Exact number is not included in the table due to the small sample size; values are reported as numbers (%).

Outcomes	With malnutrition	Without malnutrition	p-value
Pancreatitis	171 (22.9%)	1196 (24.3%)	0.41
Cholecystitis	17 (2.3%)	127 (2.6%)	0.62
Cholangitis	205 (27.5%)	1076 (21.2%)	<0.05
Sepsis	237 (31.8%)	718 (14.6%)	<0.05
Hemorrhage	28 (3.8%)	102 (2.1%)	<0.05
Intestinal perforation	*	11 (0.22%)	

Odds ratios of clinical outcomes and corresponding p-values after adjusting for age, sex, race, and the Charlson Comorbidity Index are listed in Table 3. Malnutrition was an independent risk factor for inpatient mortality (adjusted odds ratio (aOR) 2.54, 95% confidence interval (CI): 1.70-3.82, p < 0.05), sepsis (aOR 2.20, 95% CI: 1.82-2.65, p < 0.05), hemorrhage (aOR 1.64, 95% CI: 1.05-2.56, p < 0.05), and intestinal perforation (aOR 4.29, 95% Cl:1.61-11.46, p < 0.05). However, adjusted odds ratios of pancreatitis (aOR 1.05, 95% Cl: 0.87-1.28, p = 0.61), cholecystitis (aOR 0.84, 95% CI: 0.50-1.42, p = 0.52), and cholangitis (aOR 1.04, 95% CI: 0.87-1.26, p = 0.66) were not statistically significant.

**Table 3:** Multivariate regression analysis of clinical outcomes. *Adjusted for age, sex, race, and the Charlson Comorbidity Index; CI, confidence interval.

Outcomes	Adjusted odds ratio* (95% CI)	p-value
Pancreatitis	1.05 (0.87-1.28)	0.61
Cholecystitis	0.84 (0.50-1.42)	0.52
Cholangitis	1.04 (0.87-1.26)	0.66
Sepsis	2.20 (1.82-2.65)	<0.05
Hemorrhage	1.64 (1.05-2.56)	<0.05
Intestinal perforation	4.29 (1.61-11.46)	<0.05
Inpatient mortality	2.54 (1.70-3.82)	<0.05

## Discussion

This study indicates that malnutrition is associated with higher risks for sepsis, hemorrhage, and intestinal perforation in patients undergoing inpatient ERCP. These worse outcomes can undoubtedly contribute to the higher mortality, length of stay, and total hospital cost, as shown in this study. These results are consistent with previous studies showing the association between malnutrition and increased mortality, length of stay, and hospital charge [9,10]. However, it is also possible that the higher length of stay may have contributed to the higher hospital charge, which was not further investigated in this study.

Although this study shows an overall increased risk of sepsis, the odds ratios of common ERCP-associated infections such as cholangitis and cholecystitis were not statistically different between the groups with and without malnutrition. Cholangitis may develop in the setting of incomplete biliary drainage and stent obstruction or migration, and cholecystitis is thought to be from gallbladder contamination by nonsterile contrast material [14]. The pathogenesis of these outcomes does not seem to be significantly affected by malnutrition, so the increased prevalence of sepsis in the group with malnutrition may be caused by the suppressed immune system associated with malnutrition [8]. Malnutrition impairs cellular immunity by causing atrophy of the thymus, lymph nodes, and tonsils, resulting in decreased CD4 and secretory immunoglobulin A, and weakened delayed hypersensitivity and phagocytosis [15]. Thus, further analysis of types of infection that are more prevalent in the group with malnutrition may provide more insight regarding what type of infection needs to be monitored more closely and if prophylactic antibiotics would be beneficial to cover the specific infection to improve the outcomes in patients with malnutrition.

Bleeding related to ERCP is most commonly associated with sphincterotomy and may happen in about 0.3-2.0% of cases [14]. Malnutrition is known to increase bleeding risk as micronutrients such as vitamin K, vitamin C, copper, and zinc play essential roles in coagulation, and adequate absorption is required [16-19]. Although malnutrition itself has not been identified as a risk factor for ERCP-related bleeding in previous studies, the American Society for Gastrointestinal Endoscopy (ASGE) recommends coagulation studies before endoscopy in patients who have malnutrition [14,20]. This study illustrates the association between malnutrition and increased bleeding risk related to ERCP and further emphasizes the importance of following the ASGE recommendation.

Post-ERCP perforation occurs in approximately 0.4% of the cases [21]. Malnutrition results in poor skin integrity, such as diminished tensile strength, nutrient delivery, underlying fat, and dermal thickness, which can explain the higher risk of perforation in the group with malnutrition [22]. Although malnutrition was not previously recognized as a risk factor for post-ERCP perforation, this study shows the association between malnutrition and post-ERCP perforation, which warrants additional studies to evaluate the potential causal relationship.

A retrospective study involving 44 million adult inpatient cases at 460 sites illustrated that oral nutritional supplements reduced length of stay, cost, and readmission risk [23]. However, a significant proportion of patients do not receive their calculated goal calories throughout their hospital stay due to multiple reasons such as waiting for the return of bowel sounds [6]. The worse outcomes of ERCP associated with malnutrition in this study further emphasize the need for prompt identification of malnutrition in hospitalized patients and optimizing their nutritional status before ERCP.

The data used in this study are obtained from the NIS database, which relies on billing code inputs provided by healthcare providers. Thus, inaccurate billing may result in either a lower or higher number of diagnoses found in the study. In addition, as it only contains inpatient data, delayed outcomes after discharge are not captured. Indication, approach, and the number of attempts of ERCP can alter the outcomes, which also cannot be identified. Nonetheless, the NIS database provides a large sample size to estimate patient characteristics and outcomes at a national level, which is its greatest strength.

## Conclusions

As malnutrition is common among hospitalized patients, patients who undergo inpatient ERCP may have malnutrition. These patients are at increased risk for worse outcomes, such as increased inpatient mortality, sepsis, hemorrhage, and intestinal perforation. Understanding the potential outcomes of inpatient ERCP in malnourished patients and a thorough evaluation of the patient’s nutritional status to assist with risk stratification for ERCP may ultimately improve outcomes of ERCP in this population.
